# Supporting Parental Decision-Making After Life-Limiting Fetal Diagnoses: The Role of Perinatal Hospice and the NOVA-L Decision Support System

**DOI:** 10.3390/healthcare14111516

**Published:** 2026-05-29

**Authors:** Margherita Dahò

**Affiliations:** Department of Psychology, Educational Science and Human Movement, University of Palermo, Viale delle Scienze, Ed. 15, 90146 Palermo, Italy; margherita.daho@unipa.it

**Keywords:** artificial intelligence, comfort care, decision support system, decision-making, general psychology, human–computer interaction, life-limiting condition, palliative care, perinatal grief, hospice

## Abstract

**Background**: Prenatal diagnosis of life-limiting fetal conditions often leads to counseling focused primarily on therapeutic abortion. Perinatal hospice has emerged as an alternative model of care for families who choose to continue the pregnancy. This paper has two primary aims. First, it discusses structured perinatal hospice programs and their role in supporting parental decision-making after such diagnoses, with attention to ethical and emotional complexities. Second, the paper introduces NOVA-L (Navigating Options & Vital Assistance for Life-limiting conditions), a conceptual Decision Support System (DSS) designed to complement perinatal hospice care. **Methods**: The paper provides a conceptual and descriptive analysis of the Comfort Care clinical model. It also outlines the proposed architecture of NOVA-L. DSSs combine clinical guidelines, research data, and outcome registries on digital platforms, providing evidence-based information and AI-supported analytical tools. Their potential adaptation to perinatal hospice care is explored. **Results**: The Comfort Care model involves interdisciplinary counseling, structured communication, and psychosocial support to facilitate clarification of parental values and care pathways. NOVA-L is presented as a complementary tool that may enhance transparency in risk evaluation and option comparison through accessible interfaces under professional supervision. **Conclusions**: Structured perinatal hospice programs may enhance clarity and compassion in decision-making. The conceptual integration of AI-supported DSS tools, such as NOVA-L, could strengthen ethically grounded, emotionally sensitive parental support.

## 1. Introduction

Decision-making is a central component of human cognition, shaping everyday actions and complex professional judgments [1]. Individuals routinely make decisions ranging from simple preferences to high-stakes medical choices that involve uncertainty and risk. In this sense, decision-making represents a form of problem-solving, especially in ambiguous contexts such as healthcare, where consequences are ethically and emotionally significant. Effective decision-making, therefore, requires not only selecting among alternatives but also understanding context, weighing competing values, integrating uncertain information, and anticipating outcomes [1]. Two main frameworks for conceptualizing decision-making are the normative and naturalistic models. Normative models promote structured, rational approaches, such as decision matrices or cost–benefit analyses, assuming that individuals aim to maximize expected benefits subject to defined constraints [2]. In contrast, naturalistic models examine decisions as they unfold in real-life contexts, highlighting the influence of emotions, prior experiences, time pressure, and situational complexity [3,4]. From this perspective, people often rely on heuristics and narratives rather than purely quantitative evaluation. Integrating these approaches offers a framework that is analytically rigorous yet attentive to lived experience. This integration is particularly relevant in perinatal hospice, where medical uncertainty intersects with ethical reflection and profound emotional involvement [5].

In England, more than 86,000 children live with serious medical conditions that may shorten their lifespan [6]. Some illnesses, such as Leigh’s disease, are progressive and inevitably fatal, whereas others, including certain cancers, may respond to treatment but carry substantial risk [6]. Genetic conditions such as anencephaly or trisomy 13 and 18 are associated with limited life expectancy, typically days or months, although longer survival is documented [7]. Advances in medical care, including home ventilation and improved management of complications, have extended survival for some children [7]. However, these developments also generate complex ethical and emotional challenges for families and healthcare professionals. One of the earliest and most difficult decisions arises at prenatal diagnosis, when parents must decide whether to continue the pregnancy. In cases of severe life-limiting conditions, therapeutic abortion is frequently presented as an option. Some hospitals, however, also provide perinatal hospice programs that support families who choose to continue the pregnancy through coordinated medical guidance, psychological support, birth planning, palliative care, and bereavement services [8,9,10]. These programs aim to ensure that decisions are informed, supported, and aligned with parental values.

This paper discusses how structured perinatal hospice programs assist parents throughout the demanding decision-making journey following a prenatal diagnosis of a life-limiting condition, with particular attention to ethical and emotional complexities (Section 2 and Section 3). In this context, the Comfort Care unit is presented in Section 4 as an example of a model that integrates structured information with empathetic support to foster clear, compassionate decision-making. The paper then outlines the general framework of Decision Support Systems (DSS) in healthcare and palliative care [11,12] (Section 5). Finally, it introduces NOVA-L (*Navigating Options & Vital Assistance for Life-limiting conditions*), a conceptual DSS designed to complement perinatal hospice care (Section 6). By presenting information through accessible interfaces, such systems may help families and clinicians more clearly evaluate risks and care pathways. NOVA-L thus illustrates how technology can enhance structured, emotionally sensitive decision support in perinatal hospice.

## 2. Decision-Making During Adverse Prenatal Diagnosis and Grief

Parental identity often begins at conception, as individuals start to imagine themselves as caregivers and construct mental representations of their future child; as pregnancy progresses, these representations become increasingly concrete and emotionally invested [13]. A negative prenatal diagnosis can abruptly disrupt this developmental trajectory, confronting parents with the loss of the imagined child to whom they have already bonded [14]. Such news frequently provokes intense emotional reactions, including shock, disbelief, sadness, anger, guilt, shame, or disappointment, fundamentally altering expectations about the future [15]. Parents may struggle to reconcile their hopes with the medical reality, particularly in cases of life-limiting conditions where the infant’s lifespan may be limited to minutes, hours, or weeks. In these circumstances, healthcare professionals typically present several options, including therapeutic abortion, which effectively interrupts the anticipated transition to parenthood [9]. This option is often considered medically safer and, in some cases, less emotionally burdensome, as it may reduce prenatal attachment and potentially mitigate aspects of grief [13]. Historically, practices such as separating parents from stillborn infants were justified as protective measures aimed at minimizing trauma [16]. However, contemporary evidence suggests that parental adjustment and decision-making depend on multiple interacting factors, including the degree of acceptance of the child’s condition and its limitations. Not all parents can navigate this emotional turmoil effectively; some may resort to defensive coping strategies, such as denial or minimization, to manage overwhelming distress [17]. Feelings of anger may be directed toward themselves, the medical team, the illness, or even the child, and additional strain may arise when siblings are involved, as parents must also support them and explain the anticipated loss or shortened life expectancy of a brother or sister [18].

Struggling to accept a child’s illness can impair coping capacities, adaptive functioning, and cognitive clarity [17]. Intense affective states, such as guilt, anger, or profound sadness, may cloud judgment, heighten anxiety or depressive symptoms, and trigger ruminative thinking [19,20,21]. A growing body of research demonstrates that emotions significantly influence decision-making processes, e.g., [3,4,22], sometimes orienting choices toward immediate emotional relief rather than long-term reflection. At the same time, the capacity to cope with loss and make ethically complex decisions is shaped by internal and external resources, including personality traits, resilience, problem-solving skills, self-compassion, and social support [23]. Within this context, deciding whether to terminate a pregnancy can constitute a profound moral dilemma [24], often occurring under conditions of uncertainty and emotional overwhelm. This process may also take place before the loss of the imagined child has been fully processed. Parents may subsequently question their decision and grieve not only the pregnancy itself but also the anticipated future and their emerging parental identity, thereby experiencing a multilayered mourning process [25]. The need for sensitive emotional and decisional support is therefore critical. Parental grief following the loss of a child, irrespective of age or cause, is deeply traumatic [26]. When inadequately processed, it may contribute to relational strain, including increased divorce rates [27] and couple violence [28], as well as anxiety, depression, post-traumatic stress disorder, obsessive–compulsive disorder, complicated grief, hallucinations, and other mental health conditions [29,30]. Unresolved perinatal grief may also affect subsequent relationships and parenting, hampering secure attachment to later-born children or complicating future pregnancies [31,32,33]. Finally, decision-making in this context is further complicated by inherent clinical uncertainty, particularly when prognostic outcomes are probabilistic and time-dependent [34], as well as by the unpredictability of disease trajectories in medicine [35]. These uncertainties can make it difficult for both healthcare workers and families to interpret risk, compare care pathways, and anticipate future scenarios, often increasing decisional burden and emotional distress [36].

## 3. Comprehensive Care Options for Life-Limiting Prenatal Diagnoses

In light of these emotional, cognitive, and ethical challenges, a structured discussion of the care options available following a life-limiting prenatal diagnosis is essential. Presenting therapeutic abortion as the sole option following a life-limiting prenatal diagnosis risks overlooking the profound emotional, psychological, and ethical complexities that characterize parental decision-making in this context [9,37,38]. Clinical guidance should therefore encompass a broader spectrum of possibilities, tailored to families’ values, beliefs, and coping resources, and delivered through open, transparent, and empathetic communication [39,40]. Some parents may wish to continue the pregnancy but remain unaware that perinatal hospice represents a viable and structured alternative. Offering multiple, compassionately framed options can promote informed choice, reduce perceived coercion, and support healthier psychological adjustment over time [38,39].

Evidence suggests that when termination is not positioned as the only pathway, many parents elect to continue the pregnancy. In a seven-year longitudinal study of perinatal hospice practice involving 72 families facing life-limiting fetal diagnoses, Korzeniewska-Eksterowicz et al. [40] found that 68 couples chose to carry the pregnancy forward. Among these, 47 pregnancies resulted in live births and 21 in intrauterine fetal demise, illustrating both medical uncertainty and the substantial decisional burden involved. This prevalence of continuation underscores the strength of parental commitment even under severe prognostic circumstances. Continuing the pregnancy may allow time for bonding, memory-making, and emotional preparation for loss [8,41]. It affirms the intrinsic value of the child’s life, however brief, and respects parents’ desire to cherish meaningful moments [9]. Perinatal hospice care provides coordinated, interdisciplinary collaboration support to families expecting a child with a life-limiting condition [42,43], aiming to enhance quality of life, ensure comfort and dignity, and accompany families through pregnancy, birth, and, when applicable, postnatal palliative care until natural death. Such programs also foster an environment in which family members can cope with anticipatory grief while creating lasting memories [8,43,44]. In some cases, early delivery may represent an additional medically guided option.

From a psychological perspective, structured and compassionate assistance significantly facilitates parental readjustment. A “good care” approach, initiated at diagnosis and sustained throughout the clinical pathway, is strongly associated with improved long-term well-being [15,45,46,47,48,49]. Such care fosters trust, clear communication, and shared decision-making. International guidelines further emphasize the importance of offering parents the opportunity for physical contact with their deceased child, as this can facilitate grief processing [16,50,51]. Empirical studies indicate that mothers who hold their stillborn infants often report fewer symptoms of anxiety and depression than those who decline this opportunity [52,53]. Physical contact renders the loss tangible and meaningful; parents frequently describe these moments as characterized by tenderness, pride, and gratitude [50,54,55,56]. Conversely, declining contact is sometimes followed by regret [50,57]. Given that the infant’s body remains warm and soft for a short period after stillbirth [58], such contact may help parents acknowledge the child’s existence in a deeply human way. Complementary rituals—religious or secular—along with preserving belongings or photographs, further support meaning-making and symbolic bonding [44,56,59,60]. Even when parents initially decline interaction, healthcare professionals often safeguard mementos, recognizing the enduring parental bond formed during pregnancy [50]. Within this spectrum of care options, perinatal hospice models such as Comfort Care provide a practical example of interdisciplinary collaboration support for families facing life-limiting prenatal diagnoses, as further discussed in Section 4. These programs are generally grounded in shared core principles, including family-centered care, symptom management, and psychosocial support [9,10,61]. However, their implementation may vary across healthcare systems in terms of organizational structures, resources, and clinical integration pathways. Furthermore, most available evidence originates from high-income settings, highlighting a gap in implementation research from low-resource contexts [62].

## 4. Comfort Care: An Example of Perinatal Hospice

**Figure 1:**
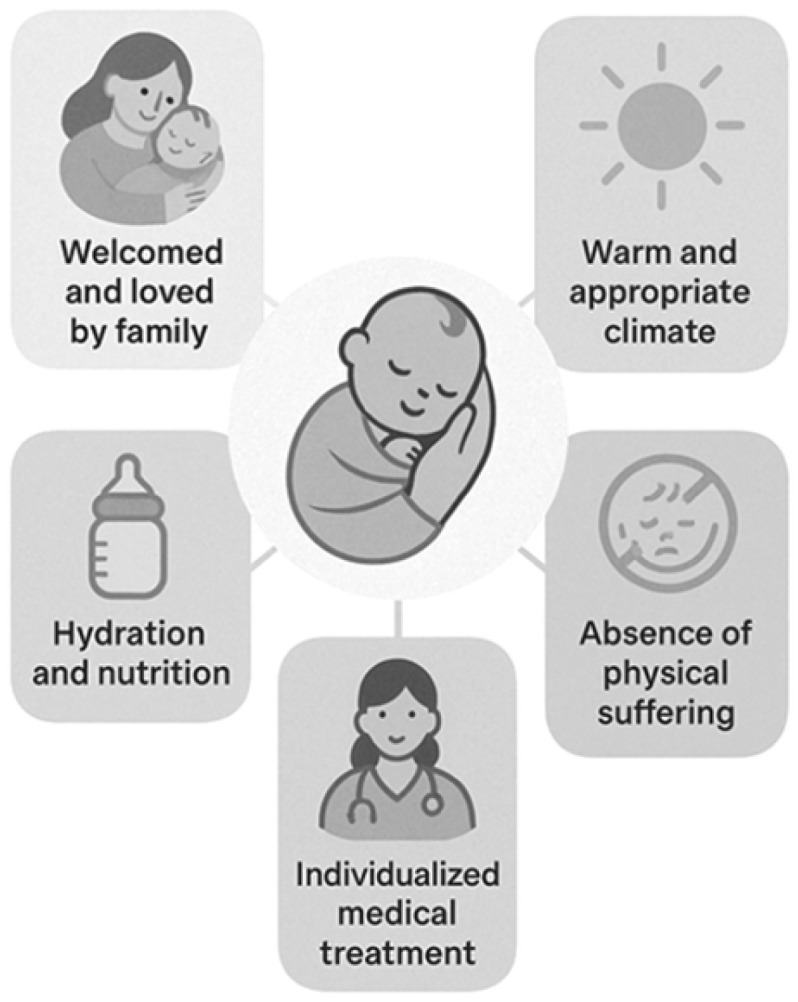
Essential needs in Comfort Care for neonates in the NICU.

Comfort Care is a specialized medical and nursing approach designed to ensure the well-being of newborns with life-limiting conditions due to severe congenital malformations or incurable genetic anomalies [40]. These infants often survive only minutes, hours, or, at most, a few months. However, the approach also applies to extremely preterm infants or those affected by irreversible infections, sometimes diagnosed in utero [10]. Within Neonatal Intensive Care Units (NICUs), multidisciplinary teams, typically composed of neonatologists, nurses, psychologists, social workers, and child life specialists, collaborate to provide integrated medical and emotional support to both infants and families. These teams play a central role in guiding parental decision-making following severe prenatal diagnoses, offering a protected environment and the resources necessary for informed, values-based choices. Additional services, such as volunteer networks and professional photographers, foster a compassionate atmosphere and help preserve meaningful family memories. The primary objective of Comfort Care in the NICU is to ensure the newborn’s comfort and well-being until death, addressing fundamental needs. These include being welcomed and loved by their family, adequate hydration and nutrition, appropriate environmental conditions, relief from physical suffering, and individualized medical care tailored to the diagnosis [9,63]. While these needs may seem basic, their fulfillment requires the full expertise and coordination of many providers, given the fragility and complexity of these patients. These essential needs are summarized in Figure 1, which illustrates the core domains of Comfort Care. Parents are encouraged to remain with their child at all times, except in exceptional circumstances such as medical emergencies or public health restrictions. To foster intimacy and presence, chairs are placed beside incubators or beds. When the newborn’s death is imminent, families may be invited to the Comfort Care Room, a dedicated, private space ensuring tranquility, privacy, and emotional support during the final moments.

Kangaroo Care, or skin-to-skin contact, is central in fostering parent–infant bonding, regulating infant temperature, and promoting feelings of safety and affection [64]. When parental presence is not possible, trained staff provide physical comfort through gentle holding to ensure warmth and serenity. For infants who survive beyond the first hours, appropriate nutrition and hydration via breastfeeding, bottle-feeding, or nasogastric administration are essential, with family participation encouraged to maintain the parent–infant bond established during pregnancy. Modern NICU technology supports these goals: incubators recreate intrauterine-like conditions, most diagnostic procedures (e.g., blood tests, imaging, sensory screenings) can be performed in situ to minimize stress, and low-intensity lighting protects retinal health, supports circadian rhythms, and promotes neurological stability [65,66]. End-of-life comfort is ensured through effective pain management, guided by validated clinical scales and integrated palliative care protocols, to minimize suffering and preserve dignity [67].

When a severe disease or malformation is diagnosed prenatally, the care team meets with the parents in the Comfort Care Room. During this meeting, the team discusses whether to continue the pregnancy, explains how to support the baby’s comfort, and guides informed decision-making and emotional support [9]. The primary physician fosters trust by acknowledging the parental bond, clearly explaining the diagnosis, detailing Comfort Care, and clarifying each team member’s role. Parents also receive literature with reflections, guidance, and stories from other families, e.g., [68,69], as well as a personalized *Birthing Plan*, a document that helps parents plan and organize the birth and postnatal care according to their preferences. It specifies delivery choices, medical and post-natal care, end-of-life procedures, and any cultural or religious rituals, ensuring that the family’s wishes are respected and that the experience is as meaningful and supportive as possible [70]. Follow-up meetings provide ongoing emotional support and medical updates, integrating new decisions into the Birthing Plan.

After birth, the neonatologist confirms the diagnosis if necessary and ensures the baby’s comfort while facilitating family bonding. The medical and nursing teams monitor the baby’s condition and manage pain, while psychologists, social workers, and child life specialists provide additional support to family members. Parents may receive an empty “*Memory Box*” to collect mementos, such as locks of hair, hand and footprints, photos, cards, or sibling drawings, affirming the baby’s presence and love while offering a tangible source of remembrance [44]. This time is filled with joy and celebration, with the baby often remaining in the parents’ arms until the baby passes in a loving environment. Farewells may include religious or special rituals to aid the grieving process, and counselors or social workers assist families before and after death, often attending funerals to provide ongoing support. The bonds established frequently endure beyond the baby’s passing and can positively influence subsequent pregnancies.

## 5. Decision-Support System in Palliative Care

Building on the discussion of parental experiences and structured support models, several systemic and organizational challenges remain that may limit the consistent delivery of high-quality perinatal palliative care. For instance, despite growing interest in perinatal hospice programs, parents often report insufficient support from society, family, and healthcare providers both during pregnancy and after the loss [71,72,73]. Supporting decision-making for parents of children with life-limiting conditions is crucial but often overlooked. Many healthcare professionals may be unaware of perinatal hospices, particularly in less developed countries, underscoring the need to expand access to these services [74]. One strategy to improve parental support is the use of DSSs, computer-based tools that integrate data, analytical models, and user-friendly interfaces to aid complex decision-making [11]. DSSs are widely applied in sectors such as business, finance, logistics, and healthcare, enabling organizations to make better decisions by leveraging data and analytics [12]. In healthcare, DSSs can operationalize clinical guidelines by integrating them with patient-specific data to generate personalized, context-sensitive recommendations that consider prognosis, individual preferences, and anticipated quality of life. Evidence also suggests that these systems enhance nursing performance, strengthen adherence to protocols, improve situational awareness, and contribute to overall care quality [75]. For example, Beacon DSS consolidates multiple structured oncological data sources, including clinical guidelines, clinical trial repositories, and outcome registries, into a single, secure platform that enables clinicians, researchers, and policymakers to access and query information efficiently [76]. Rather than generating automated treatment recommendations, Beacon DSS serves as a curated evidence platform, providing validated, reliable, and comparable information to help reduce disparities in care [77,78,79].

Although DSSs are more developed in oncology, they hold significant promise in palliative care, including perinatal contexts [80]. Increasingly, these systems are powered by AI techniques that enhance predictive accuracy, personalization, and adaptive clinical guidance. AI-driven DSSs support complex decisions related to symptom management, care planning, and end-of-life treatment by integrating large datasets, identifying clinically relevant patterns, and generating context-sensitive outputs [81]. Beyond workflow optimization, AI-enabled DSSs can improve clinicians’ knowledge of palliative care options, support patient-centered care, systematically track patient status, and generate automated alerts for timely interventions [81]. Illustrative examples include *ePAL* (Electronic Palliative Care DSS) [75], which leverages structured symptom questionnaires and monitoring tools to improve pain management and deliver personalized recommendations. *Aleph PC* (Aleph Palliative Care) [82] is a more advanced AI application: a web-based machine-learning system that integrates multidimensional patient data, including functional indices, comorbidities, laboratory values, and diagnostic parameters, to generate predictions of mortality and frailty. Notably, Aleph PC incorporates explainability mechanisms, increasing transparency and clinician trust in algorithmic outputs. Another example is *PreCare* (Advance Care Planning Assistant) [83]. This AI-enabled web application guides patients and proxies through value clarification, knowledge acquisition, and structured decision-making, thereby enhancing preparedness for end-of-life discussions. Collectively, these systems illustrate how AI can move beyond static, rule-based architectures toward adaptive, data-driven, and explainable decision-support frameworks in palliative care.

Many other DSS platforms offer symptom monitoring, allowing rapid responses to changes in patient condition and facilitating systematic presentation of care options to clinicians, patients, and families. AI-supported decision-support systems may be particularly valuable in resource-limited settings where specialist access is constrained. However, the adoption of such systems remains limited due to organizational, technical, and training-related barriers [82,84]. Successful implementation requires institutional support, integration with electronic health records, secure infrastructure, and trained staff [11]. Data privacy and security are particularly critical when handling sensitive information about caregivers’ choices, health conditions, and end-of-life preferences [84]. DSS recommendations must also remain transparent and explainable, so that clinicians, patients, and caregivers can understand how suggestions are generated and trust the system [84]. Without these conditions, DSSs risk being underutilized or distrusted, limiting their real-world impact. The integration of AI within DSS offers additional benefits, including rapid analysis of large datasets, detection of subtle patterns, and continuous learning from new data; yet, at the same time, it requires careful review by multidisciplinary healthcare teams to ensure safe, ethical, and reliable decisions, as they may generate inaccurate or invented information [85].

## 6. NOVA-L: A Tailored DSS for Families in Perinatal Hospice Contexts

**Figure 2:**
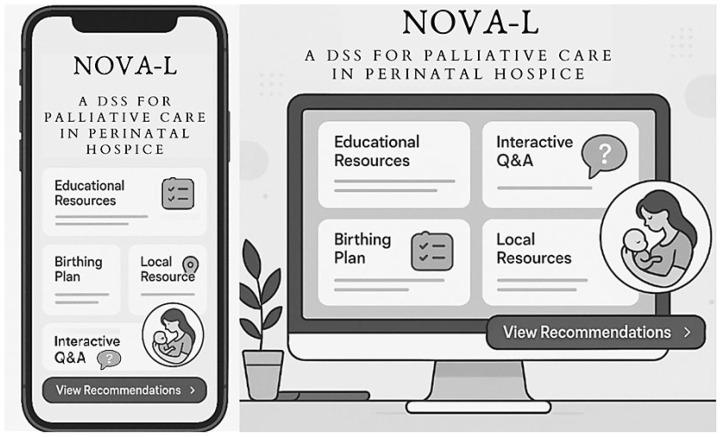
A conceptual illustration of a DSS for Palliative Care in Perinatal Hospice, shown in both laptop and mobile formats.

Despite the increasing use of DSS in adult palliative care, e.g., [75,82,83], there is a notable absence of tools specifically designed for parents of children with life-limiting conditions. Drawing on the author’s experience in perinatal hospice care and digital DSS, NOVA-L (*Navigating Options & Vital Assistance for Life-limiting conditions*) is proposed as a conceptual, family-centered DSS intended to empower parents, facilitate informed decision-making, and complement clinical care (Figure 2). At present, no fully developed prototype exists; NOVA-L represents a novel idea still under development, with efforts underway to secure funding for its creation. Inspired by the idea of a “nova,” a new, bright star symbolizing guidance, hope, and the preciousness of life, NOVA-L envisions the integration of evidence-based knowledge, compassionate guidance, and awareness. In addition, the platform envisions the responsible integration of AI methods to enhance personalization, data organization, and adaptive information delivery. Rather than replacing clinical judgment, AI components would serve as supportive analytical layers, helping tailor content to family needs while maintaining transparency and professional oversight.

The system is designed to assist parents in navigating the extraordinarily complex and emotionally charged decisions inherent to perinatal hospice care. Crucially, NOVA-L does not make or suggest moral or clinical decisions. It functions as an interpretive and organizational tool, aggregating validated medical, psychosocial, and ethical data and presenting them in accessible, interactive formats to support reflection and informed discussion, while keeping all interpretations and choices mediated by clinicians. NOVA-L is conceptualized as a continuation of existing healthcare DSS models and offers a range of features outlined in the following sections. These features are organized into four core functional modules: (Section 6.1) educational resources and interactive guidance; (Section 6.2) development of a digital birthing plan; (Section 6.3) access to local resources; and (Section 6.4) decision-making support through scenario visualization and interactive decision trees. Finally, Section 6.5 and Section 6.6 discuss recommendations for integrating the tool into clinical care, as well as future directions and ethical considerations for the development of the full platform.

### 6.1. Educational Resources and Interactive Guidance

The system will provide families with comprehensive, accessible, and verified information about care pathways, palliative interventions, comfort-focused therapies, symptom management strategies, and potential outcomes. Educational content could include multimedia resources such as short videos demonstrating comfort techniques, infographics summarizing treatment options, and case narratives from other families to enhance understanding. For example, a video tutorial could show how Kangaroo Care supports bonding and helps regulate an infant’s temperature, while a companion infographic could illustrate recommended symptom-management steps.

In addition, an interactive Q & A interface will allow parents to seek clarification and receive personalized responses in real time, particularly when healthcare professionals are not immediately available. These resources aim to support parents’ knowledge and emotional readiness, helping them anticipate challenges, prepare questions for consultations, and feel more confident in decision-making from birth planning to postnatal care. Guidance may also include evidence-based information about coping strategies, grief processing, and communication with extended family, reinforcing both practical and emotional support.

### 6.2. Digital Birthing Plan

The digital Birthing Plan module is another feature that enables parents to document detailed preferences for the birth setting, delivery method, comfort measures, end-of-life care, and any religious or cultural rituals. Beyond recording choices, it may include prompts and examples to help families reflect on their priorities, consider contingency plans, and communicate their wishes clearly to the care team. For instance, the module could feature short video testimonies of other parents describing creative ways they celebrated their baby’s life, such as organizing a small welcome party, creating a professional photo book, or even uniting two beds so the whole family could stay together and cuddle the newborn.

The module can also be available in printable or shareable versions for consultations with clinicians, midwives, or palliative care staff, ensuring that parental preferences are consistently understood and respected. Integration with educational content and scenario simulations may allow families to explore “what-if” scenarios, helping them anticipate emotional and logistical implications of different decisions.

### 6.3. Access to Local Resources

The directory could connect families to a broad range of local and virtual resources, including nearest perinatal hospice programs, counseling and mental health services, peer support networks, spiritual care providers, and practical assistance such as transportation, financial aid, or legal guidance. Resources may be curated to include verified contacts, service descriptions, and ratings or reviews to support informed selection.

Families will filter resources by location, availability, language, or service type, enabling them to build a personalized support network that meets their clinical, emotional, and logistical needs throughout the perinatal journey. For example, parents could find information on nearby parking options for long hospital stays, affordable local accommodation for family members, or bereavement support services. They might also find volunteer services for meal delivery, childcare for siblings, or familiar spiritual counselors who can provide practical guidance to ease daily burdens and allow them to focus on their newborn.

### 6.4. Decision Support and Scenario Visualization

**Figure 3:**
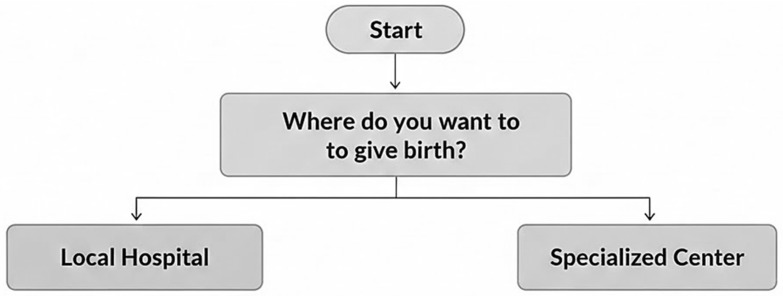
Example of an interactive decision tree: Birth location. This decision tree illustrates how families can explore birth-setting options interactively. At each step, the system poses a question (e.g., “Where do you want to give birth?”) and presents multiple options (the number of options may vary by question, countries, and local norms). In this case, a list of potential clinics would appear. Based on the family’s selections, subsequent questions appear to illustrate practical, logistical, and emotional considerations. Importantly, this tool is purely reflective and educational: selecting an option does *not* create real-world outcomes, but it helps parents anticipate implications, explore scenarios, and support discussions with healthcare professionals.

Interactive decision trees, scenario simulations, and visual care timelines are usually present in DSS [11]. In NOVA-L, they will allow families to compare care pathways, for instance, between local and specialized centers, or in-person and virtual counseling. AI-driven analytical tools may integrate evidence-based datasets, clinical guidelines, and contextual inputs (e.g., gestational age, diagnosis category, available local resources) to generate adaptive scenario visualizations. In addition, machine learning models could support pattern recognition across anonymized outcome registries, enabling the system to present probabilistic projections in a transparent and interpretable format. Importantly, these outputs would remain explanatory rather than prescriptive, serving as decision-support simulations rather than automated recommendations. An example of a decision tree for birth location is illustrated in Figure 3, which shows how families can explore options, anticipate practical considerations, and integrate personal preferences with clinical guidance.

### 6.5. Integration of NOVA-L into Clinical Care: A Few Recommendations

As usual, healthcare professionals first meet with parents to explain the diagnosis, discuss available options, and answer any questions. Rather than being introduced during the initial consultation, when parents may still be experiencing acute emotional shock and struggling to process additional information, the system could be presented in a subsequent encounter, once emotional arousal has decreased, and families are better able to engage with supportive resources. Parents can thus review educational content, scenario simulations, and decision-support features at their own pace, update or annotate their digital Birthing Plan with new preferences, and bring these insights to follow-up consultations. Importantly, registered parents can also access dedicated phone lines for real-time support, maintaining privacy and confidentiality.

### 6.6. Future Directions and Ethical Considerations

Beyond individual support, NOVA-L is envisioned as a platform to raise broader public awareness of neonatal life-limiting conditions, enabling a more informed understanding, reducing misconceptions, and fostering empathy. This is in line with evidence suggesting that digital health tools and decision-support systems can enhance health literacy and improve access to and comprehension of health information [86,87]. Future directions include not only developing a functional prototype but also rigorously evaluating the platform’s usability, acceptability, and feasibility. This process will involve iterative user testing, quantitative and qualitative evaluations, and co-design sessions with key stakeholders, including families and healthcare professionals. Particular attention will be given to user interactions with the interface, as usability and user engagement are key determinants of effective implementation [11]. The perceived clarity and emotional sensitivity of the content will also be evaluated, given their importance in supporting decision-making under conditions of uncertainty and emotional distress. In addition, the practicality of integrating the tool into routine care pathways will be assessed. Additionally, future studies may explore cross-cultural validation and multilingual adaptation to ensure that NOVA-L remains relevant and accessible across diverse healthcare systems. Importantly, healthcare systems differ not only culturally and organizationally but also in their regulatory and legal frameworks [62]. The implementation and availability of perinatal palliative care and related decision-support tools may also be influenced, indeed, by national and regional regulatory frameworks, which can shape the range of legally available care options and affect how decision-making pathways are structured across healthcare systems [62]. For these reasons, the initial implementation will likely proceed gradually, starting in one to two European countries where perinatal hospice programs are already well established, to ensure regulatory alignment, clinical feasibility, and contextual adaptation before broader international expansion. Finally, future developments may expand NOVA-L’s functionalities in line with existing palliative DSS models (e.g., PreCare, ePAL), extending its use beyond parental support to provide broader access to clinical guidelines, protocols, and care standards for other stakeholders involved in perinatal care. Such efforts will help guarantee that the platform is not only technically sound but also meaningful, usable, and supportive for those who rely on it.

The NOVA-L knowledge base will be implemented as a structured, searchable repository integrating standardized clinical guidelines and validated evidence sources, using controlled vocabularies and expert-curated data pipelines to ensure consistency, traceability, and clinical interoperability. From a technical perspective, it incorporates explainable AI (XAI) frameworks to ensure that algorithmic outputs remain transparent, traceable, and clinically interpretable [88]. Techniques such as feature attribution methods (e.g., SHAP or LIME), rule-based hybrid models, and model-agnostic interpretability layers are used to clarify how specific clinical variables (e.g., gestational age, diagnostic indicators, and prognostic uncertainty) influence recommendations. Robust validation procedures, including external dataset testing and continuous performance monitoring, will be required to assess reliability, calibration, and generalizability across diverse populations.

Ethical safeguards will also constitute a foundational component of system design. First, AI systems can reproduce or amplify existing social and healthcare inequities [89]. Therefore, bias detection and mitigation strategies must address potential disparities related to socioeconomic status, cultural background, disability perspectives, and institutional practices. Particular attention should also be devoted to preventing automation bias, ensuring that AI outputs do not unduly influence parental autonomy or clinician judgment [89]. Second, transparent data governance protocols, including secure data storage, informed consent procedures, audit trails, and compliance with applicable data protection regulations, will be essential given the highly sensitive nature of perinatal decision-making contexts. Stringent privacy-preserving mechanisms, including data minimization, pseudonymization, and double access control, will be implemented to safeguard sensitive patient and family information throughout the system lifecycle, in line with established privacy-by-design principles in digital health and AI systems [90]. Finally, human-in-the-loop architectures are envisioned as a structural requirement rather than an optional feature [91]. In this model, healthcare professionals retain interpretative authority, contextualizing AI-generated insights within the broader clinical, psychosocial, and ethical landscape of each case.

## Data Availability

No new data were created or analyzed in this study.

## Abbreviations

The following abbreviations are used in this manuscript:

DSS	Decision Support System
NOVA-L	Navigating Options & Vital Assistance for Life-limiting Conditions
NICU	Neonatal Intensive Care Unit
